# Adrenocortical adenoma with myelolipomatous metaplasia: a potential diagnostic pitfall: a case report and review of the literature

**DOI:** 10.1186/s13256-021-02937-9

**Published:** 2021-07-04

**Authors:** Mohammad Hossein Anbardar, Neda Soleimani, Saman Nikeghbalian, Maryam Mohebbi

**Affiliations:** 1 Department of Pathology, Shiraz Medical School, Shiraz University of Medical Sciences, Shiraz, Iran; 2 Department of Pathology, Shiraz Transplant Center, Abu Ali Sina Hospital, Shiraz University of Medical Sciences, Shiraz, Iran; 3 Department of hepatopancreatobiliary and organ transplant surgery, Shiraz Transplant Center, Abu Ali Sina Hospital, Shiraz University of Medical Sciences, Shiraz, Iran; 4 Department of Surgery, Shiraz Medical School, Shiraz University of Medical Sciences, Shiraz, Iran

**Keywords:** Adrenal adenoma, Adrenal myelolipoma, Myelolipomatous metaplasia

## Abstract

**Background:**

Adrenal incidentalomas are often found during investigation for another tumor or unrelated problems. Except for adrenal myelolipoma (second most common primary adrenal incidentaloma following adrenocortical adenomas), adrenal lipomatous tumors are uncommon generally and are often described as case reports in the literature. Since the amount of fat is variable, without the help of advanced imaging techniques, some adrenal lipomatous tumors may be misdiagnosed before pathologic examination. Herein, we report a case of adrenal adenoma with myelolipomatous metaplasia that was excised as a periceliac mass in the setting of recurrent pancreatic cyst.

**Case report:**

A 45-year-old Iranian woman with hypertension and end-stage renal disease presented with recurrence of a pancreatic cyst (previous pathologic report was mucinous cyst adenoma). During exploratory laparotomy, the mentioned pancreatic cyst was tightly attached to the stomach and jejunum. There was also a periceliac round rubbery lesion (firstly diagnosed by endoscopic ultrasound) that was excised for ruling out malignancy. Histologic examination of the periceliac mass was found to be adrenocortical adenoma with foci of myelolipomatous metaplasia. The pancreatic cyst histology was just a pseudocyst.

**Conclusion:**

Our case highlights the significance of complete evaluation of incidental findings before surgical intervention, even in the setting of another primary tumor. Myelolipoma and myelolipomatous change (metaplasia) are two different entities. Although very similar as to pathogenesis, there are still some differences.

## Introduction

Adrenal incidentalomas are often found during investigation for another tumor or unrelated problems. In general, most adrenal incidentalomas (70–80%) are benign adenomas that cause no symptoms. Differential diagnosis consists of pheochromocytoma, adrenocortical carcinoma, lymphoma, and metastases from various malignancies. Adrenal lipomatous tumors (myelolipoma, lipoma, teratoma, angiomyolipoma, and adrenocortical tumors with lipomatous and myelolipomatous metaplasia) should also be included [1–5]. Since the amount of fat is variable in lipomatous tumors, in the absence of advanced imaging techniques, they may be misdiagnosed as malignant lesions before pathologic evaluation [6]. Herein, we report a case of adrenal adenoma with myelolipomatous metaplasia that was excised in the setting of recurrent pancreatic neoplasm.

## Case report

A 45-year-old Iranian woman was transferred to our center to be evaluated for abdominal pain and recurrence of a pancreatic cyst. One year ago, she underwent central pancreatectomy and cholecystectomy at another center because of incidental finding of a large pancreatic cyst, and the pathologic report was benign mucinous cyst adenoma. Then, she presented with upper abdominal pain, mildly elevated carcinoembryonic antigen (CEA) level, and recurrence of pancreatic cyst, which were found on follow-up. She was a case of hypertension (HTN) and end-stage renal disease (ESRD) for 5 years due to idiopathic nephrotic syndrome, and she was taking prednisolone, metoral, and amlodipine. She was dialyzed twice a week. She was a married housekeeper lady, G2P2A0L2, without any history of smoking or alcohol consumption. She had normal menstruation, and there was not any history of abdominal cramp, polyuria, polydipsia, polyphagia, and recent weight gain. The patient’s psychologic and family history was not significant. On physical examination, blood pressure was 130/80 mmHg with normal pulse rate and temperature, 76 beats per minute and 37.1 °C, respectively. Her body mass index (BMI) was 24.5. Chest examination was unremarkable, and the abdomen was soft with no organomegaly. She had normal face and body fat distribution without any hirsutism or pink stretch mark on the abdomen and trunk. There was no significant problem on neurologic examination.

The patient had high blood urea nitrogen (BUN) and creatinine, 27 (6–20) mg/dl and 4.2 (0.6–1.3) mg/dl , respectively. Serum fasting blood sugar (96 mg/dl) and complete blood count (CBC) [white blood cell (WBC): 10.8 × 10^3^/µl, hemoglobin: 13.2 g/dl, and platelet count: 224 × 10^3^/µl] were within normal ranges. Liver enzymes including aspartate aminotransferase (AST), aspartate aminotransferase (ALT), and alkaline phosphatase (ALP) were 23(3–40), 21(3–40), and 146(80–306) IU/L respectively. Abdominal sonography showed a large cystic lesion in the body and tail of the pancreas. In addition to pancreatic cyst (measuring 10 × 8 × 4 cm), endoscopic ultrasound discovered a 2 × 2 × 2 cm solid mass at the left periceliac area. Exploratory laparotomy revealed the mentioned pancreatic cyst with tight attachment to the posterior wall of the stomach and jejunum and the periceliac lesion with suspicious attachment to the left kidney. For ruling out malignancy, the specimens of distal pancreatectomy, splenectomy, and periceliac lesion excision were sent for pathologic evaluation. Gross sectioning of the pancreas showed a hemorrhagic cyst with 1 cm maximum wall thickness. The periceliac lesion was a round creamy yellow solid firm mass with heterogeneous cut surface.

**Fig. 1:**
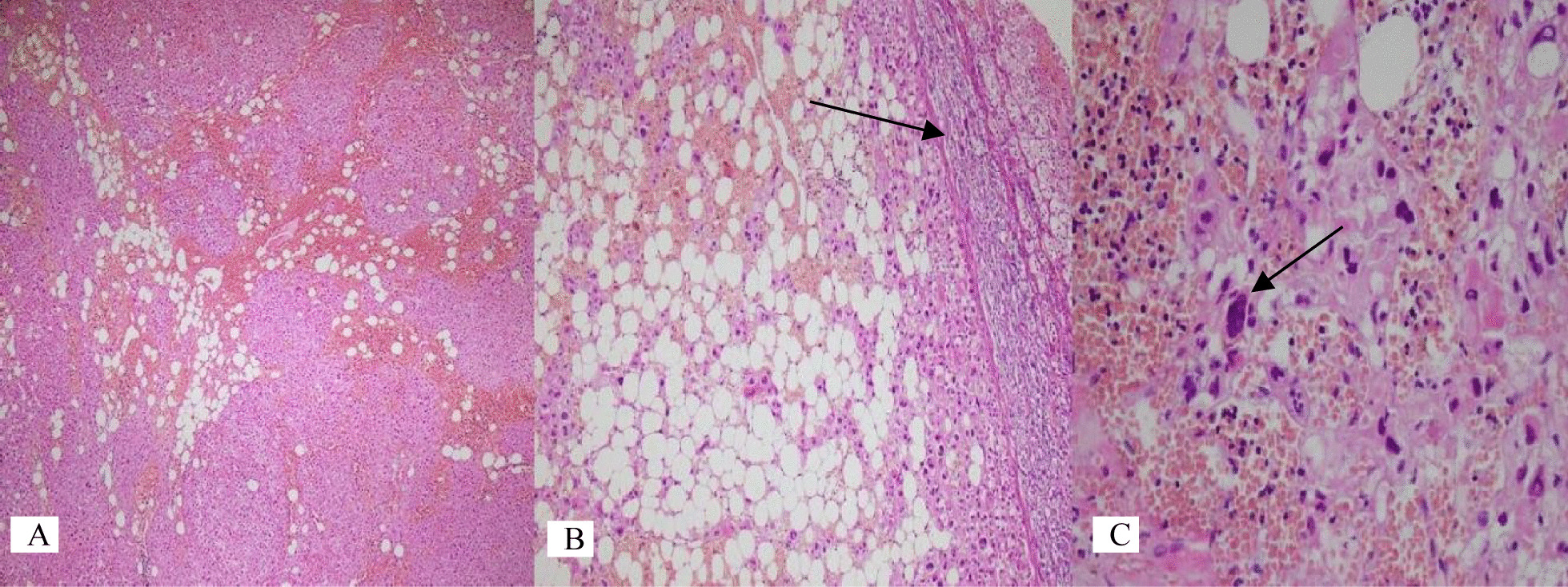
Histological examination. **A** Adrenocortical neoplasm intermixed with adipose tissue (hematoxylin and eosin ×40). **B** ACA with myelolipomatous metaplasia and a thin rim of normal adrenal gland (hematoxylin and eosin ×100). **C** Myelolipomatous metaplasia with bone marrow elements including megakaryocyte (arrow) (hematoxylin and eosin ×400)

**Fig. 2:**
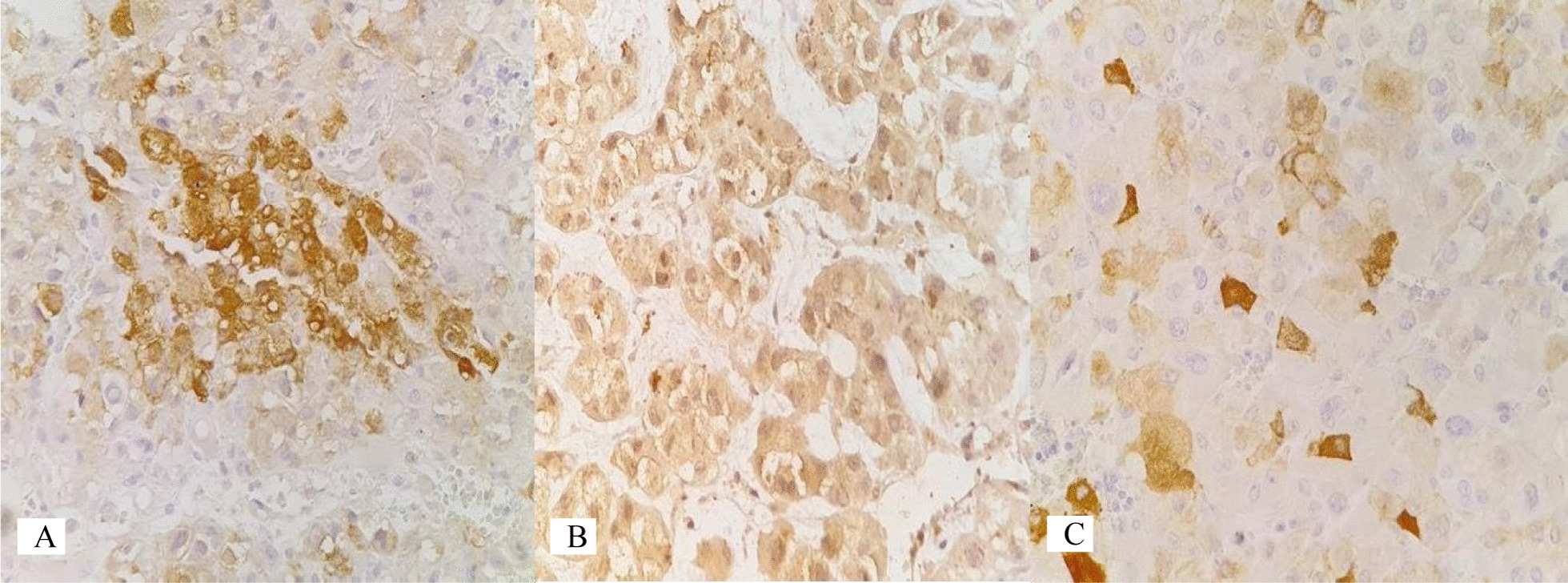
Immunohistochemistry study. **A** Inhibin ×400. **B** Melan A ×400. **C** Synaptophysin ×400

**Fig. 3:**
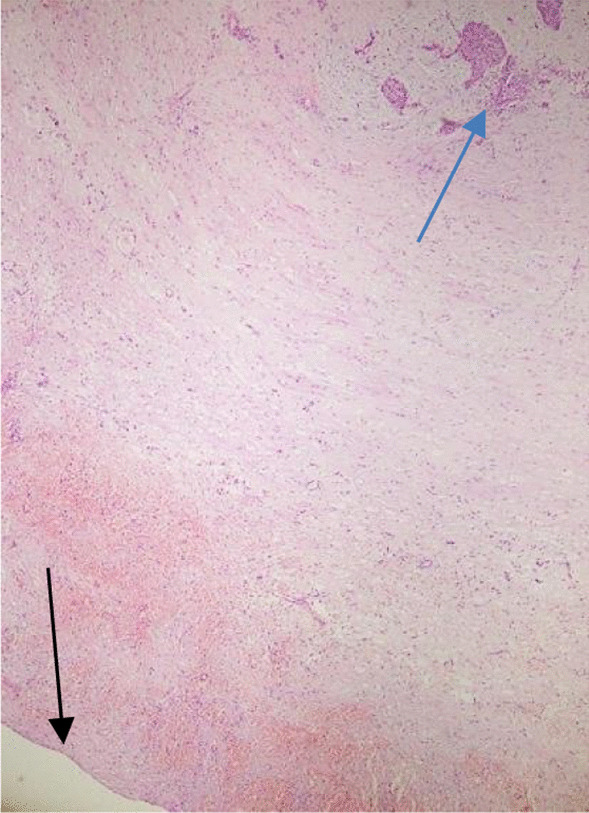
Microscopy of the pancreas cyst showing no definite epithelial lining (black arrow) and remnant of the endocrine component (blue arrow)

On microscopy, sections of the periceliac mass showed an adrenocortical adenoma (ACA) with multifocal areas of adipose tissue admixed with bone marrow elements (including megakaryocytes), features in keeping with ACA (approved by immunohistochemistry) with myelolipomatous change, instead of malignancy (Figures 1, 2). The pancreatic cyst histology was just a pseudocyst (Figure 3). During hospital admission, the patient received the previously prescribed medication with the same dosage in addition to a short-acting benzodiazepine (oxazepam 10 mg per night)as a sedative. In a follow-up visit 2 weeks later, the patient showed improvement in her symptoms, and during the 10-month follow-up visits, no clinical or radiologic deficits were noted. We requested and obtained the informed consent from the patient for publishing the case report and the publication of the accompanying images. Also, our institutional approval was not required to publish the case details.

## Discussion

Our patient was a middle-age woman with multiple chronic diseases (ESRD and HTN), recurrent cyst of the pancreas, and a new periceliac mass, which was incidentally found by endoscopic ultrasound during post-partial pancreatectomy follow-up. Since the previous pathology report of the pancreas was consistent with mucinous neoplasm and regarding the malignant potential of mucinous cystic lesions, it was considered as a suspicious malignant lesion. Fortunately, on pathologic examination, the periceliac mass was an adrenal gland showing ACA associated with myelolipomatous metaplasia. During the 10-month follow-up visits, no related symptom or abnormal radiologic findings were noted.

Adrenal incidentalomas are clinically silent adrenal masses that are found during the investigation of unrelated problems. Myelolipoma is a lipomatous tumor, which is the second most common primary adrenal incidentaloma following ACAs, accounting for about 3% of the incidentalomas [6, 7]. Generally, adrenal lipomatous tumors are uncommon neoplasms. Myelolipoma is the predominant type, but other types of lipomatous adrenal gland tumors such as lipomas, teratoma, angiomyolipoma, and adrenocortical tumors with fat component (lipomatous and myelolipomatous metaplastic changes) are often described as case reports in the literature [8, 9]. Among these lipomatous lesions, just myelolipoma and myelolipomatous metaplasia contain bone marrow elements, in addition to adipose tissue.

Adrenal myelolipoma was first described by Arnold as “adrenal lipoma” in 1866, but it was Gierke in 1905 who described the myeloid component. The term “myelolipoma” was coined by Oberling in 1929. Most of the early studies described it as an incidental autopsy finding or as occasional case reports. Despite its benign behavior, adrenal myelolipoma is clinically relevant as it might cause significant difficulties in differential diagnosis of other adrenal tumors [9, 10]. Myelolipomatous metaplasia is a secondary degenerative change in a primary adrenal tumor. It usually occurs in ACA, which is the most common adrenal tumor. Due to increased availability, access to and use of abdominal imagery investigations [ultrasound scan, computed tomography (CT) scan and magnetic resonance imaging (MRI) scan] for the evaluation of various conditions and symptoms, cases of adrenal lipomatous tumors are now diagnosed more frequently [11].

In terms of pathogenesis, it seems that chronic adrenal stimulation, such as necrosis or inflammation due to cancer, Cushing’s disease, HTN, diabetes, obesity, and stressful lifestyle could lead to metaplasia of the reticuloendothelial cells, which could lead to the development of adrenal myelolipomas. This hypothesis is supported by the increased incidence of the lesion in the advanced years of life. Another hypothesis claims that adipocytes develop from the mesenchymal stem cells in the endothelium; this results in inflammation causing the adrenal cortex to secrete mediators responsible for the recruitment of hematopoietic progenitors. The myelolipomatous metaplasia also occurs in response to stress related to stimuli such as necrosis, infection, stress, or trauma [9, 12–15]. The metaplastic change in our case could be explained by chronic stress of ESRD, HTN, and the previous surgery.

Generally, there are no specific laboratory tests that would be diagnostic of adrenal myelolipoma, and in contrast to ACA, which could be functional or nonfunctional, most myelolipomas are silent. The National Institute of Health Consensus Panel on adrenal incidentaloma (2002) concluded that adrenal myelolipoma could be regarded as an exception to the mandatory metabolic work-up of a newly discovered adrenal mass [8–10]. It also makes sense that adrenal tumors with myelolipomatous metaplasia show metabolic changes that are compatible with the primary tumor [15–17].

Ultrasound is usually inadequate for the diagnosis of myelolipoma. On CT and MRI, the diagnosis of myelolipoma can be confidently made when an adrenal mass is composed of at least 50% fat. In larger masses with lesser amounts of fat, lipomatous or myelolipomatous degeneration of other adrenal tumors should be considered. On the other hand, the typical imaging features of adenoma in CT and MRI include small size, homogeneous and well-circumscribed, presence of intracellular lipid, and low-to-intermediate signal intensity on T2-weighted images. When ACAs show atypical features (that is, mixed areas of low and higher attenuation or heterogeneous signal intensity), the possibility of an adrenal collision tumor or secondary degenerative changes should be considered [10, 18].

On pathologic examination, it is easier to distinguish myelolipoma from an adrenocortical tumor with myelolipomatous metaplasia. In fact, myelolipoma is a well-demarcated combination of fat and myeloid tissue, while myelolipomatous metaplasia is usually present as scattered ill-defined foci of fat and myeloid tissue within another tumor.

In management of adrenal incidentalomas, European Society of Endocrinology Clinical Practice Guideline in collaboration with the European Network for the Study of Adrenal Tumors recommend determining if an adrenal mass is benign or malignant at the time of initial detection, which is just possible through CT scan and MRI study. Patients with an asymptomatic, nonfunctioning unilateral adrenal mass and obvious benign features on imaging studies do not need surgical intervention, whereas adrenalectomy has been suggested in patients with unilateral adrenal masses with radiological findings suspicious of malignancy (heterogeneous features and size > 4 cm) [19].

Until now, a few adrenocortical tumors showing myelolipomatous metaplasia with full separated demographic and pathologic data have been reported (Table 1).

**Table 1:** Demographic and pathologic data of adrenal tumors with myelolipomatous metaplasia

No.	Authors	Age	Sex	Adrenal pathology	Underlying disease	Other remarkable finding
1	Vyberg *et al*. [15]	31	F	ACA	–	–
2	Parenteau *et al*. [16]	28	M	Pigmented adrenal hyperplasia	–	–
3	Lamas *et al*. [17]	49	F	ACA	–	–
4	Lamas *et al*. [17]	67	F	ACA	HTN/DM	–
5	Montone *et al*. [20]	50	F	Adrenocortical neoplasm of UMP	–	–
6	Montone *et al*. [20]	56	F	Adrenocortical neoplasm of UMP	–	–
7	Montone *et al*. [20]	67	F	ACA	HTN	–
8	Inomoto *et al*. [21]	46	F	Black ACA	HTN	–
9	Yamada *et al*. [22]	72	F	ACA	Chronic hepatitis C	Concurrent endothelial cyst in adrenal
10	Shastri *et al*. [23]	40	F	Oncocytoma	HTN	Bilateral
11	Gurbuz *et al*. [24]	66	M	ACA	Goiter surgery Nephrectomy	–
12	Yokoyama *et al*. [25]	34	F	ACC	–	Tumor site was ectopic adrenal (retroperitoneum)
13	Chandramahanti *et al*. [26]	66	F	ACA	HTN/DM	–
14	Hendry *et al*. [27]	60	F	ACA	HTN/hypothyroidism	Concurrent epithelioid angiosarcoma in adrenal
15	Kinebuchi *et al*. [28]	77	M	Cavernous hemangioma	History of gastric and rectal cancer	–
16	Dokmetas *et al*. [29]	53	F	ACA	HTN/DM	Concurrent schwannoma in adrenal
17	Larose *et al*. [30]	61	F	Macronodular adrenal hyperplasia	–	Bilateral
18	Fırat *et al*. [31]	62	F	ACA	–	–
19	Fırat *et al*. [31]	73	M	ACA	HTN	Concurrent endothelial cyst in the adrenal
20	Current case	45	F	ACA	HTN ESRD	Concurrent pancreas cyst

*ACA* adrenocortical adenoma, *ACC* adrenocortical carcinoma, *UMP* uncertain malignant potential, *HTN* hypertension, *DM* diabetes mellitus, *ESRD* end-stage renal disease

Although myelolipoma and myelolipomatous metaplastic change are very similar in pathogenesis, there are still some fine differences that help us make a more accurate diagnosis. Table 2 presents a comparison between myelolipoma and myelolipomatous metaplasia in terms of demography and pathology. The myelolipomatous metaplasia data have been extracted from our review.

**Table 2:** Demographic and pathologic differences between myelolipoma [6] and myelolipomatous metaplasia

	Myelolipoma	Myelolipomatous metaplasia^a^
Dominant sex	No gender predilection	Female predominant
Age range, years (mean)	41–84(62) [6]	28–77(52.5)
Chronic disease	HTN/DM	HTN/DM
Dominant side	Right	Left
Gross and microscopic feature	Well-demarcated macroscopic fat	Ill-demarcated microscopic fat
Most common collision tumor	ACA	ACA

*HTN* hypertension, *DM* diabetes mellitus, *ACA* adrenocortical adenoma

^a^The mentioned data are according to current review

Among 20 cases of myelolipomatous metaplasia that we reviewed, the primary adrenal pathology in 13 cases (65%) was ACA (the most common adrenal tumor). Sixteen cases (80%) were female, and just four cases (20%) were male. This sex pattern could be justified by female predominance of ACA. The mean age was a little lower in myelolipomatous metaplasia, and the dominant side was left in contrast to myelolipoma (most cases are right sided). Common chronic diseases were HTN and DM in both groups. Myelolipoma is usually a well-defined mass with fat component that is easily seen grossly, while myelolipomatous metaplasia is an ill-defined microscopic finding. In addition to adrenocortical hyperplasia, adrenocortical carcinoma, adrenocortical neoplasm of uncertain malignant potential, and oncocytoma, some metaplastic changes were accompanied with vascular lesions such as endothelial cyst (two cases), cavernous hemangioma, and epithelioid angiosarcoma.

Adrenal tumors with myelolipomatous change may show heterogeneous appearance and could mimic malignancy even using advanced radiologic studies. Although in our case no further radiologic evaluation was done, regarding the recurrence of pancreatic cyst, the histology of the primary cyst, and also surgery protocols in approach to suspicious adrenal incidentalomas, finally there was no change in the patient treatment, but it is very important to consider incidentalomas before operation to reduce surgical complications and it should be in the differential diagnosis when we deal with multiple tumors.

## Conclusion

In a patient with a primary tumor, considering any new mass as malignancy should be avoided. Our case highlights the significance of complete evaluation of incidental findings by laboratory tests and radiologic studies before surgical intervention, even in the setting of another primary tumor. Based on observations in this case report and to the best of our knowledge, myelolipoma and myelolipomatous change (metaplasia) are two different entities. Although very similar considering pathogenesis, there are still some differences. In terms of epidemiologic and pathologic features, it appears that myelolipomatous metaplasia follows the primary adrenal tumor more than myelolipoma.

## Data Availability

All data generated or analyzed during this study are included in this published article.
